# Proposed design for the PGAA facility at the TRIGA IPR-R1 research reactor

**DOI:** 10.1186/2193-1801-2-597

**Published:** 2013-11-09

**Authors:** Bruno T Guerra, Radojko Jacimovic, Maria Angela BC Menezes, Alexandre S Leal

**Affiliations:** Centre for Development of Nuclear Technology (CDTN), Brazilian Nuclear Energy Commission (CNEN), Av. Antonio Carlos 6627 CAMPUS UFMG, CEP, 31270-901 Belo Horizonte, Brazil; Jožef Stefan Institute, Jamova cesta 39, 1000 Ljubljana, Slovenia; Department of Nuclear Engineering (DEN), Federal University of Minas Gerais (UFMG), Av. Antonio Carlos 6627, CEP, 31270-901 Belo Horizonte, Brazil

**Keywords:** PGAA facility, Neutron activation analysis TRIGA IPR-R1, CDTN (Centre for Development of Nuclear Technology), PGAA (Prompt Gamma Activation Analysis), NAA (Neutron Activation Analysis)

## Abstract

**Background:**

This work presents an initial proposed design of a Prompt Gamma Activation Analysis (PGAA) facility to be installed at the TRIGA IPR-R1, a 60 years old research reactor of the Centre of Development of Nuclear Technology (CDTN) in Brazil. The basic characteristics of the facility and the results of the neutron flux are presented and discussed.

**Findings:**

The proposed design is based on a quasi vertical tube as a neutron guide from the reactor core, inside the reactor pool, 6 m below the room’s level where shall be located the rack containing the set sample/detector/shielding. The evaluation of the thermal and epithermal neutron flux in the sample position was done considering the experimental data obtained from a vertical neutron guide, already existent in the reactor, and the simulated model for the facility.

**Methods:**

The experimental determination of the neutron flux was obtained through the standard procedure of using Au monitors in different positions of the vertical tube. In order to validate both, this experiment and calculations of the simulated model, the flux was also determined in different positions in the core used for sample irradiation. The model of the system was developed using the Monte Carlo code MCNP5.

**Conclusion:**

The preliminary results suggest the possibility of obtaining a beam with minimum thermal flux of magnitude 10^6^ cm^-2^ s^-1^, which confirm the technical feasibility of the installation of PGAA at the TRIGA IPR-R1 reactor. This beam would open new possibilities for enhancing the applications using the reactor.

## Introduction

**Figure 1:**
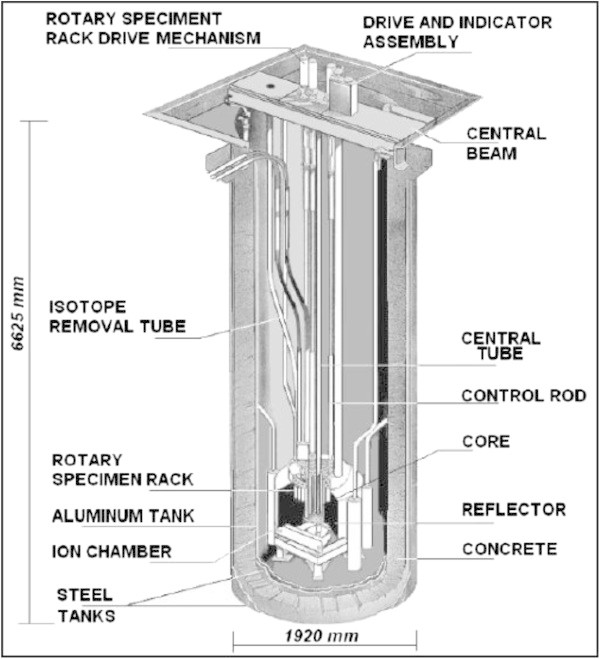
**The TRIGA IPR Mark 1 research reactor (courtesy of Dr. A. Z. Mesquita).**

The TRIGA Mark I IPR-R1 research reactor of the CDTN is operating since 1960. It is a pool type reactor cooled by natural circulation. Since 2009, the core configuration has 63 fuel elements of an alloy of uranium and zirconium hydride (20% ^235^U). The reactor operates at 100 kW but the core configuration allows the increasing of the power up to 250 kW. Figure 1 shows the reactor pool, Zangirolami et al. (2010).

**Figure 2:**
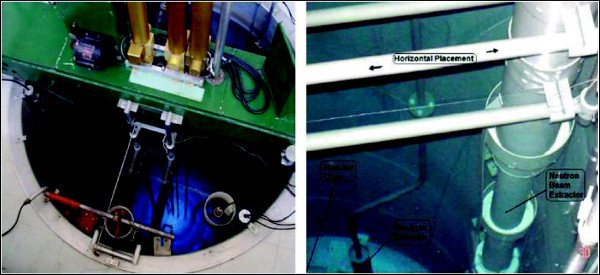
**Top view from the pool and of the vertical tube of the TRIGA reactor Amorim** 1987 **.**

During its 52 years of operation, the TRIGA has been used mainly for the training of nuclear-plant operators, neutron activation analysis (NAA), production of radioisotopes and educational activities. In the 80’s, a vertical tube, which can be shifted from the wall of the pool to a position close to the core, Figure 2, was installed for experiments of neutron radiography, but this project was soon cancelled and the tube has remained unexploited since then, Amorim (1987).

In 2001, the CDTN started a program for the optimisation of the TRIGA´s utilisation. Since then, several new projects were initiated like the implementation of the k_0_-standardization method; the investigation of the mechanisms involved for the improvement of the Brazilian gemstones and production of new radioisotopes for radiopharmaceutical studies, Leal et al. (2006a, 2006b; 2007, Leal et al. (2013), Gonçalves et al. (2011), Soares et al. (2012). For allowing the enhancement of the reactor´s utilisation, a study of raising the power of the reactor from 100 to 250 kW was already concluded and it is now in the final process of licensing.

**Figure 3:**
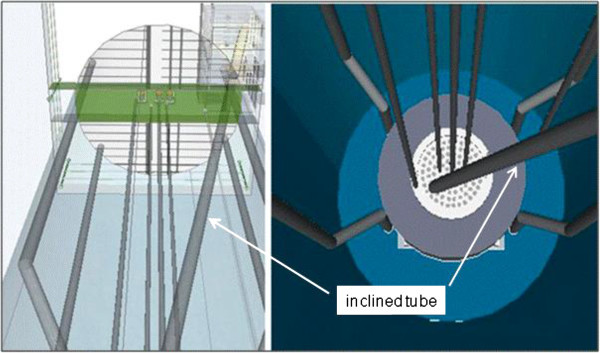
**View from the core (left) and from the room level (right), of the inclined tube (neutron beam).**

To enhance the reactor utilisation depends on the availability of a neutron beam from the reactor core, also enabling the use of the facility for PGAA measurements to be used in several fields of research, technology and industrial applications such as: geology, chemistry, materials science, archaeology, safety and security and environment among others. The PGAA is an indicated method of analysis used when the gamma radiation emitted by the activated nuclides must be detected “promptly”, it means, simultaneously, with the irradiation of the sample by neutrons. It is specially the case of some light elements as H, C, N and B. For that, it is necessary a special arrangement of the set sample-detector-shielding, as illustrated in Figure 3. For allowing the best possible sensitivity a maximum ratio signal/background is required. In spite of the low power of the reactor and the impossibility of building an horizontal neutron beam, the broad applicability of the PGAA encouraged us to evaluate the feasibility of installing an upward directed neutron beam from the reactor core. Even a low thermal neutron flux ≅ 10^6^ cm^-2^.s^-1^ would make possible PGAA with reasonable sensitivity, Leal et al. (2011), Molnar (2004).

This work describes the suggested set-up for the PGAA facility, the computational model of the system and the reactor core and the experimental data of the neutron flux.

## Availability and requirements

### Preliminary design of the PGAA facility

**Figure 4:**
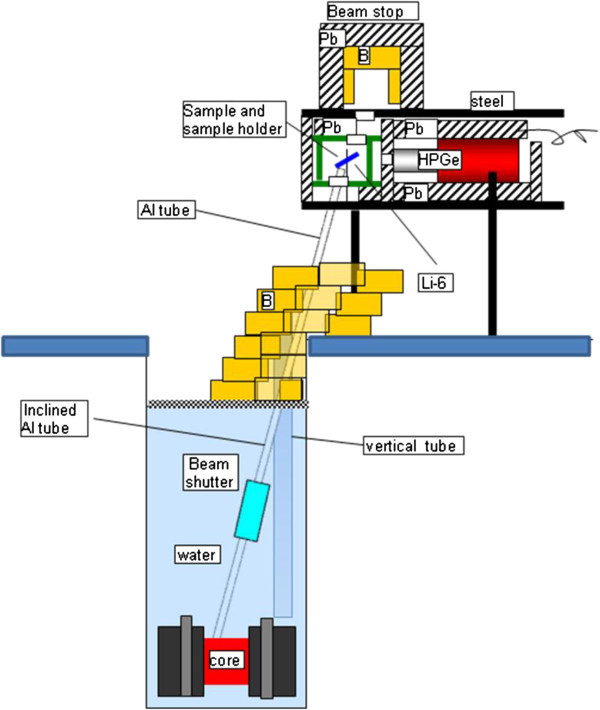
**Proposed design (inclined tube and components) for the PGNAA facility at CDTN.**

**Figure 5:**
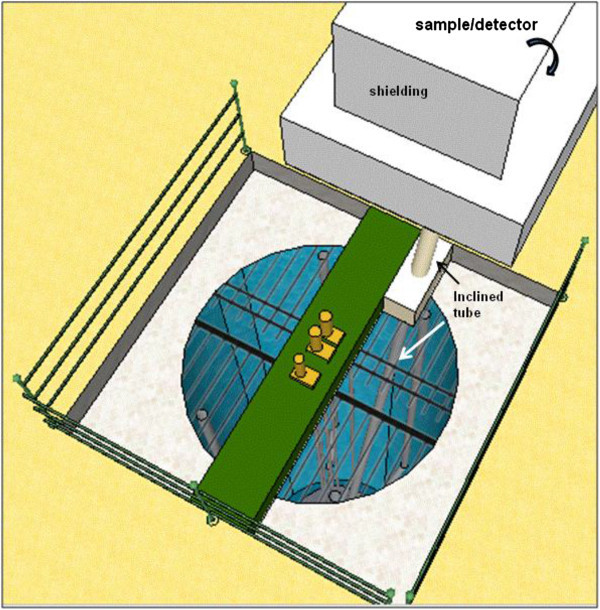
**Top view from the pool, the inclined tube and the rack with sample/detector.**

The proposed model for a PGNAA system at the TRIGA reactor is presented in Figures 3, 4 and 5. The inclined tube is a necessary alternative to avoid the heavy structure of the rack containing the shielding, the sample and the detector, be located directly over the reactor pool, which is not allowed for security reasons. The system is composed by the following main parts, Revay (2008):

- **Guide tube.** An aluminium tube with a diameter of 5 cm guides the neutron from the top of the reflector to the sample chamber. The beam will be shut flooding the tube with water. The upper part of the tube will be covered with lead shielding.
- **Sample chamber.** The beam is guided into the sample chamber from below. The chamber is made of at least 5-cm-thick lead. It has to be lined with a shielding material containing Li (with some percentage of Li-6).
- **Detector.** A 20–30% relative efficiency, n-type HPGe detector will be purchased. It will be completely covered with lead shielding leaving with just a small hole in the direction of the sample (Collimated detector). The sample chamber and the shielding of the detector is practically one box that has a collimator (a wall with a hole) in the middle.
- **Beam-stop.** The beam-stop is above the sample line. It will have an inner layer containing boron and hydrogen.

### Computational model

The Monte Carlo programs MCNP5 and MCNPX were used for modelling the design of the system and calculations of the neutronic parameters to evaluate the feasibility of installing the PGNAA at the TRIGA reactor, Dalle (2005). The MCNP computer code was used in the calculation of the reaction rates and fluxes. MCNP is a general-purpose, continuous-energy, generalized-geometry Monte Carlo transport code. The calculations reported in this paper were performed with version 5.1.40 of the code and with the ENDF/B-VII.0 cross-section library (processed at the National Nuclear Data Centre at Brookhaven, obtained from the Radiation Safety Information Computational Centre at Oak Ridge), Briesmeiter (1997).

**Figure 6:**
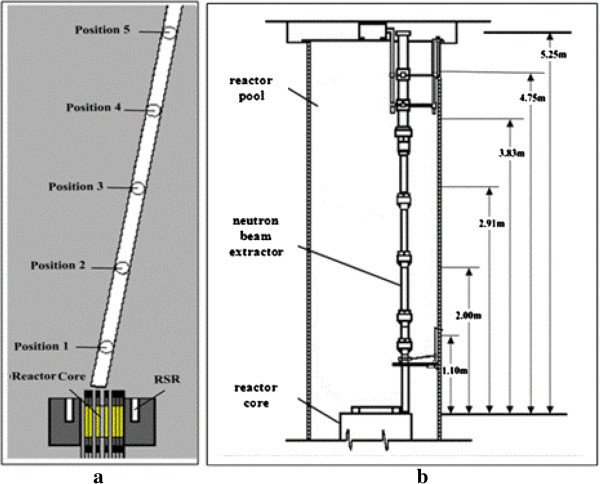
**Model of inclined tube (left) and vertical neutron beam (right).**

The thermal (E < 0.625 eV) and epithermal (0.625 eV < E < 100 keV) neutron fluxes in vertical tube were calculated and compared with experimental values in order to validate and adjust the computational model. Fast neutrons (E > 100 keV) were not investigated. The thermal and epithermal neutron fluxes in the proposed inclined tube were calculated in several positions from the core to top, Figures 6a, 6b. The validation of the model using the flux measurements in vertical tube is a required preliminary step before the authorization for the experimental measurements using the inclined tube. In this work, the KCODE option of MCNP was set to 3.5 × 10^4^ neutron histories and 2500 cycles were used. The software run in a Core Duo 3GHz processor with 48 h of processing time, in average.

**Figure 7:**
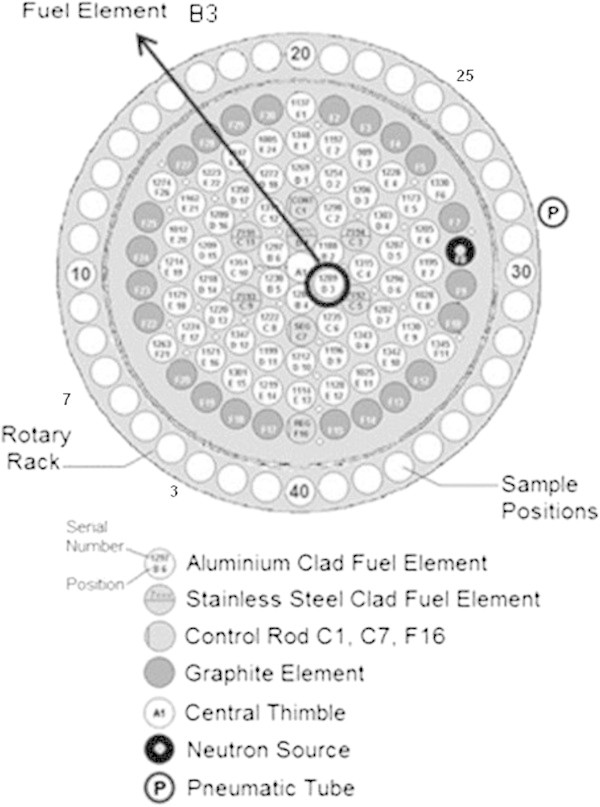
**Detailed view of the TRIGA reactor core, the carrousel and positions of irradiation.**

The model used here was based on previous studies of the reactor core, whose geometry is presented in Figure 7, Snoj et al. (2011). In order to revalidate the modification performed in the previous model of the core, the thermal neutron flux was calculated in 5 positions of the carrousel, Figure 7, and compared with experimental data.

### Experimental

#### Neutron flux in the vertical tube

Three different set of experimental data (1), (2) and (3), considered in this work. In the experiment (1), performed in the 80’s just after the installation of the tube, a metallic ^115^In foil was used as a flux monitor and the cadmium ratio, used for the determination of the epithermal flux, Parry (2003). In that time, the configuration of the reactor core was different from the present one, Figure 7, due to the replacement of some fuel elements.

In the experiments (2) and (3), bare and cadmium-covered samples of gold (Au 100%), disks of 125 mg, 1.25 cm in diameter, and 0.06 mm height were used as flux monitors. The irradiation time was 4 h and 8 h, respectively, for bare and cadmium covered samples. All the experiments were performed with the reactor operating at 100 kW. After the irradiation, the gamma spectra of each sample was obtained using a system equipped with an HPGe detector GC 5019 and Genie 2000, v2.0 Spectroscopy software, provided by Canberra Industries, Inc. The counting time was adjusted to provide a net peak area of at least 10,000 counts for the 411.8 keV peak from the ^198^Au, Parry (2003). The thermal and epithermal flux were determined from the gamma spectra using the formalism of Høgdahl convention, De Corte (1987).

#### Flux in the carrousel

For the determination of the neutron flux in the carrousel, Al-Au(0.1%) foils (disks of 5 mm diameter and 0.2 mm thick) were irradiated in 5 different positions, as indicated in Figure 7. Samples were simultaneously irradiated for 2 h and the activity was measured using high-purity germanium detector (HPGe).

The activation reaction considered in this experiment is: ^197^Au (n,γ) ^198^Au, where 411.8 keV is the energy for the most prominent gamma ^198^Au photopeak. The cross section for the reaction ^197^Au (n,γ) is the highest for energies below 10 keV, making this reaction useful for the investigation of thermal and epithermal neutrons.

## Results and discussion

### Experimental and simulated flux in the carrousel

**Figure 8:**
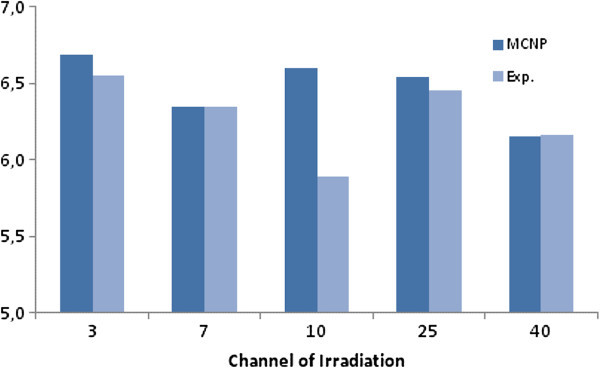
**Experimental and calculated thermal neutron flux (10** ^**11**^ **cm** ^**-2**^ **.s** ^**-1**^ **) in the five positions of the carrousel.**

Figure 8 illustrates the thermal neutron flux components in the five positions of the carrousel (channels 3, 7, 10, 25 and 40), Figure 7, and the respective values obtained by the MCNP model. The relative error (experimental/calculated) varied from 0.1 (channel 7) to 12% (channel 10). For all the positions the intrinsic standard deviation of MCNP was lower than 4% which is a satisfactory result that the standard acceptable error of MCNP is 5%, Dalle (2005). The best possible position found for the lower extremity of the inclined tube is coincident with the fuel element B3, as illustrated in Figure 7.

### Experimental and simulated flux in the vertical tube

**Figure 9:**
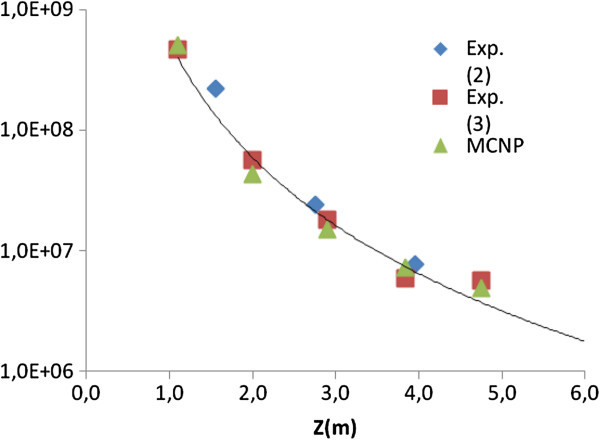
**Experimental and simulated thermal flux in the vertical channel.**

The experimental and calculated results of the thermal (ϕ_th_) and epithermal (ϕ_ep_) neutron flux, in the vertical tube, as illustrated in Figure 6b, are presented in Table 1. Figure 9 presents a comparison of the experiments (2) and (3) and the simulated model for the thermal flux.

**Table 1:** **Variation of the experimental and calculated thermal neutron flux, in cm** ^**-2**^ **s** ^**-**1^ **(**ϕ_**th**_ **), and the factor** ***f*** **(**ϕ_**th**_ **/**ϕ_**epi**_ **) in the vertical channel of TRIGA reactor with the distance Z from the core**

Z(cm)	110^(3)^	120^(1)^	155^(2)^	200^(3)^	240^(1)^	275^(2)^	290^(3)^	360^(1)^	383^(3)^	395^(2)^	475^(3)^
Experimental											
ϕ_th_	4.7 x 10^8^	5.8 x 10^7^	2.2 x 10^8^	5.6 x 10^7^	2.8 x 10^7^	2.4 x 10^7^	1.8 x 10^7^	5.2 x 10^7^	5.9 x 10^6^	7.7 x 10^6^	5.6 x 10^6^
*f*	99	112	140	58	80	---	63	73	41	---	59
Calculated (MCNP)											
ϕ_th_	5.1 x 10^8^	----	1.6 x 10^8^	4.3 x 10^7^	----	2.0 x 10^7^	1.5 x 10^7^	----	7.2 x 10^6^	6.3 x 10^6^	4.9 x 10^6^
Δ_cod._(%)	1	----	3	4	----	6	6	----	8	8	10
Δ_exp_(%)	9	----	27	23	----	17	17	-----	22	18	13
*f*	41	----	----	28	----	----	22	----	21	----	28
Δ_rel_(%)	59	----	----	51	----	----	65	----	48	----	53

^(1)^Cd-ratio method with In foil.

^(2)^and ^(3)^Cd-ratio method with Au foil.

The relative error of the simulation compared to the experimental data, (Δ_exp_), varies from 9 to 27% and the MCNP intrinsic code error, (Δ_cod_), is lower than 10%. The code error can be improved, but it requires an upgrade of the computational resources or a huge time of processing, one week or more, which is not feasible. The (Δ_exp_) is due to the conjugated effects of the intrinsic code error and the experimental error together. The variation of the thermal flux, higher than it could be expected for the considered positions, is due to the uncertainties in several factors: thickness of the Au monitor and cadmium capsule, different efficiency of the used detectors, variation of the reactor flux during the experience and others. From previous data of thermal neutron flux performed periodically in the carrousel, using similar procedures, it can be attributed a maximum experimental error of 10%, Zangirolami et. al. (2010).

The relative high error resulting for the factor *f* (ϕ_th_/ϕ_epi_) is due to difficulties for obtaining a more accurate experimental estimation of the epithermal flux. The evaluation of *f* parameter is important to give an idea of the thermalization of the beam. Because the neutron cross section are larger and the number of possible interfering reactions smaller, at lower energy, ideally, the neutrons for PGAA must have energy lower than the thermal level, Molnar (2004). One source of error is the irregularities in the kind of cadmium capsules used for the measurements and the uncertainty in the distance between the core and vertical tube, fulfilled by water. Small changes in the value of this distance modify dramatically the value of epithermal flux in the tube. A visual inspection of the pool and a new set of experiments are planned to improve the model of the system. The experiment (1) was not considered in the comparison in the Table 1 because the configuration of the core was different when the experiment was performed. From Figure 9, it can be observed a same behaviour for the calculated and measured thermal neutron flux from the experiments (2) and (3).

**Figure 10:**
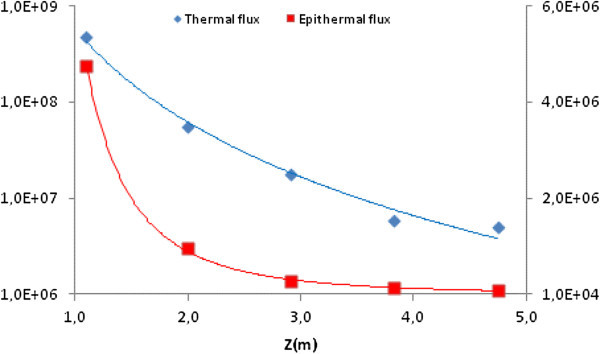
**Experimental thermal (left) and epithermal (right) flux (cm** ^**-2**^ **s** ^**-1**^ **) in the vertical channel.**

Figure 10 illustrates the behaviour of the thermal and epithermal flux obtained from the experiment (3). It can be observed that the epithermal flux decays more strongly than the thermal one, with the distance from the core. From Table 1, in the top of the vertical extractor at the floor level, the value of *f* is 59 corresponding to a ratio (epithermal/thermal) lower than 2%. This value indicates a well thermalized beam, Molnar (2004).

### Simulated flux in the inclined tube

The results calculated by MCNP of the thermal flux and the factor *f* in different positions of the inclined tube, as illustrated in Figure 6a, are presented in Table 2.

**Table 2:** **Variation of the calculated thermal neutron flux, in cm** ^**-2**^ **s** ^-1^ **(ϕ**_th_ **)**, an**d the factor** ***f*** **(ϕ**_**th**_ **/ϕ**_**epi**_ **) in the inclined tube of TRIGA’s reactor with the vertical distance Z from the core**

Z(cm)	86	178	270	361	451	541	631	721	811
ϕ_th_	4.7 x10^9^	1.1 x10^8^	2.1 x10^7^	9.5 x10^6^	5.5 x10^6^	2.9 x10^6^	1.9 x10^6^	1.4 x10^6^	1.0 x10^6^
Δ_cod._(%)	5	10	2	3	2	3	2	2	2
*f*	42	25	17	16	17	20	13	13	13

The relative error of the simulation Δ_cod_(%), is lower than 11%, which can be considered satisfactory for this preliminary model. Ideally, Δ_cod_(%), should be lower than 5%, but it requires a very high computational time due to the necessary increasing of number of neutron histories and cycles, Dalle (2005)

**Figure 11:**
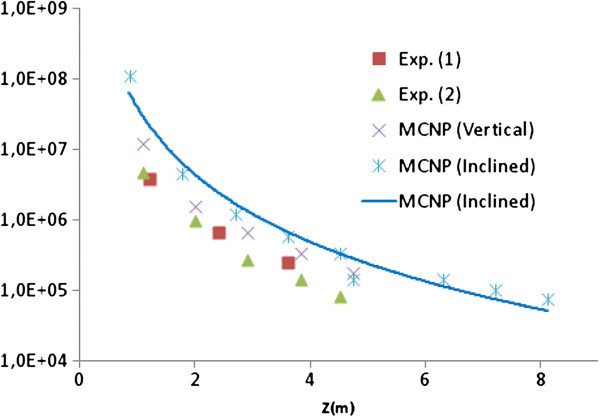
**Experimental and simulated epithermal flux (cm** ^**-2**^ **s** ^**-1**^ **) in the inclined and vertical tubes.**

The behaviour of the epithermal flux in the vertical (experimental and simulated), and the inclined (simulated) tubes is presented in Figure 11. The results suggest that epithermal flux is overestimated by the simulation, which is confirmed by the comparison of experimental and calculated *f* factor in Tables 1 and 2. Experimental data from inclined tube and the upgrade of the computational resources will allow a better estimation of the epithermal flux.

From these results, it can be expected, in the worst case, a thermal flux, ϕ_th_, of 10^6^ cm^-2^ s^-1^ in the highest possible position of the sample/detector, located approximately 3.5 m height from the reactor room level. If the set sample/detector can be positioned closer to the pool and the floor a double flux, ~2.0 × 10^6^, will be possible.

A still higher flux in the sample/detector can be obtained increasing the diameter of the tube, but in this case, the tube must be shifted for a more external position of the core, diminishing the effect of the higher diameter. This shifting also depends on mechanical adjustments of the tube with the core and the top of the pool and the configuration of the shielding. If the reactor operates at 250 kW, in the future, a thermal flux of magnitude 10^7^ can be obtained in the position of the sample/detector.

## Conclusion

The measured and calculated thermal flux in the vertical neutron beam and the simulation of a proposed inclined tube from the core in the reactor pool, confirm that the installation of a PGAA facility at the TRIGA reactor can be feasible. In the worst case, considering an inclined tube of 5 cm of diameter, a minimum neutron flux of 10^6^ would be possible in a sample/detector position located 3.5 m above the reactor room floor.

According the calculations, the flux can be increased until 10^7^, if the rack containing the set of shielding/sample/detector can be positioned closer to the reactor pool; if a tube with higher diameter can be mechanically adapted in the core and in the pool´s surface and if the reactor operates at 250 kW. A best statistic performance of simulation with a lower Δ_cod_(%), will require the improvement of the computational resources, including additional and faster processors.

Experimental measurements under way, of the neutron flux in the inclined tube and neutron and gamma dose in reactor pool will define the optimum design for the shielding and will establish an accurate minimum-maximum range of the possible neutron flux in a sample/detector set. The preliminary results present here suggest the possibility of using the reactor, at least, for some PGAA applications. Additional experimental data are necessary to make a more complete evaluation of cost/benefit.
